# ﻿*Primulinascutellifolia*, a new species of Gesneriaceae from southern Vietnam

**DOI:** 10.3897/phytokeys.187.77856

**Published:** 2021-12-13

**Authors:** Ngoc Long Vu, Tran Quoc Trung Nguyen, Gioi Tran, Quoc Dat Nguyen, Hong Truong Luu

**Affiliations:** 1 Southern Institute of Ecology and Institute of Applied Materials Science, Vietnam Academy of Science and Technology, 1D, TL29 Street, District 12, Ho Chi Minh City, Vietnam; 2 18 Hoang Quoc Viet Street, Cau Giay District, Hanoi, Vietnam; 3 Khanh Hoa Association for Conservation of Nature and Environment, Nha Trang City, Khanh Hoa Province, Vietnam

**Keywords:** Gesneriaceae, new species, *Primulina*, scutellate leaves, Vietnam

## Abstract

*Primulinascutellifolia* is described as a new species from Khanh Hoa Province, southern Central Vietnam. It is distinct in the genus in having scutellate leaves that make it a highly potential ornamental plant. The new species looks like *P.annamensis* in general shapes, sizes and colours of habit, inflorescence, flower, and leaf but is distinguishable by adaxially glabrous and abaxially strigose leaves with serrate margins, scutellate leaf blade and appressed downwards tomentose petiole, sparsely glandular hairs on apical 1/2 of the gynoecium and trapeziform one-lipped stigma with slightly emarginated apex.

## ﻿Introduction

*Primulina* Hance (Gesneriaceae) is known to have more than 220 species, with the center of species diversity in south and southwest China and North Vietnam (Weber et al. 2011; Möller and Clark 2013; Möller et al. 2016; Xu et al. 2017; Li et al. 2019; Möller 2019; IPNI 2021). As more than 40 new species have been described over the past five years, we now expect that more species will be discovered for the genus if further explorations are employed (Möller 2019; Ge et al. 2020; Tong et al. 2020; Xin et al. 2021).

In Vietnam, *Primulina* was last revised to have 23 species (Vu 2017), but this work ignored *P.crassirhizoma* F.Wen, Bo Zhao & Xin Hong (Zhao et al. 2013). Based on molecular, morphological and cytological characters, Möller et al. (2016) moved *P.cycnostyla* (B.L.Burtt) Mich.Möller & A.Weber, *P.eberhardtii* (Pellegr.) Mich.Möller & A.Weber, *P.minutihamata* (D.Wood) Mich.Möller & A.Weber and *P.tamiana* (B.L.Burtt) Mich.Möller & A.Weber to *Deinostigma* W.T.Wang & Z.Y.Li. Since then, three other new species (*P.elegans* B. M. Wang, Y. H. Tong & N. H. Xia; *P.malipoensis* L.H. Yang & M. Kang; *P.xuansonensis* W.H.Chen & Y.M.Shui) have been described from northern Vietnam (Yang et al. 2018; Chen et al. 2020; Tong et al. 2020). Therefore, prior to this paper, the total number of *Primulina* species known in Vietnam is 23.

In 2013, the last author of this paper collected a *Primulina* species in an evergreen broadleaf forest in Khanh Hoa Province. It was misidentified as *P.annamensis* (Pellegr.) Mich.Möller & A.Weber (Weber et al. 2011) – a species that grows popularly in many forests in Khanh Hoa Province and the neighbour Bidoup – Nui Ba National Park in Lam Dong Province. During our ongoing study of Gesneriaceae in southern Vietnam, we have carefully examined the plant and found that its scutellate leaves are distinct in the genus, and therefore we describe it here as a new species. Measurements of morphological characters were based on living plants whose photographs were taken with Canon EOS 7D digital camera. Morphological comparison with the close species was based on in situ observation and consultation with published literature.

## ﻿Taxonomy treatment

### ﻿ Primulina scutellifolia

Taxon classificationPlantaeLamialesGesneriaceae

Luu, N.L.Vu & T.Q.T.Nguyen sp. nov.

107D78C5-F584-5AB8-A76D-BB0C8226BFD2

urn:lsid:ipni.org:names:77234377-1

Figure 1

#### Type.

Vietnam. Khanh Hoa Province, Khanh Vinh District, Son Thai Commune, 12°11'39"N, 108°43'30"E, at ca. 1485 m elevation, 01 November 2013, *Luu Hong Truong* KH0945 (holotype SGN!; isotypes SGN!; PHH!; VNMN!).

#### Diagnosis.

*Primulinascutellifolia* differs from other congeners in having scutellate leaves.

**Figure 1.**
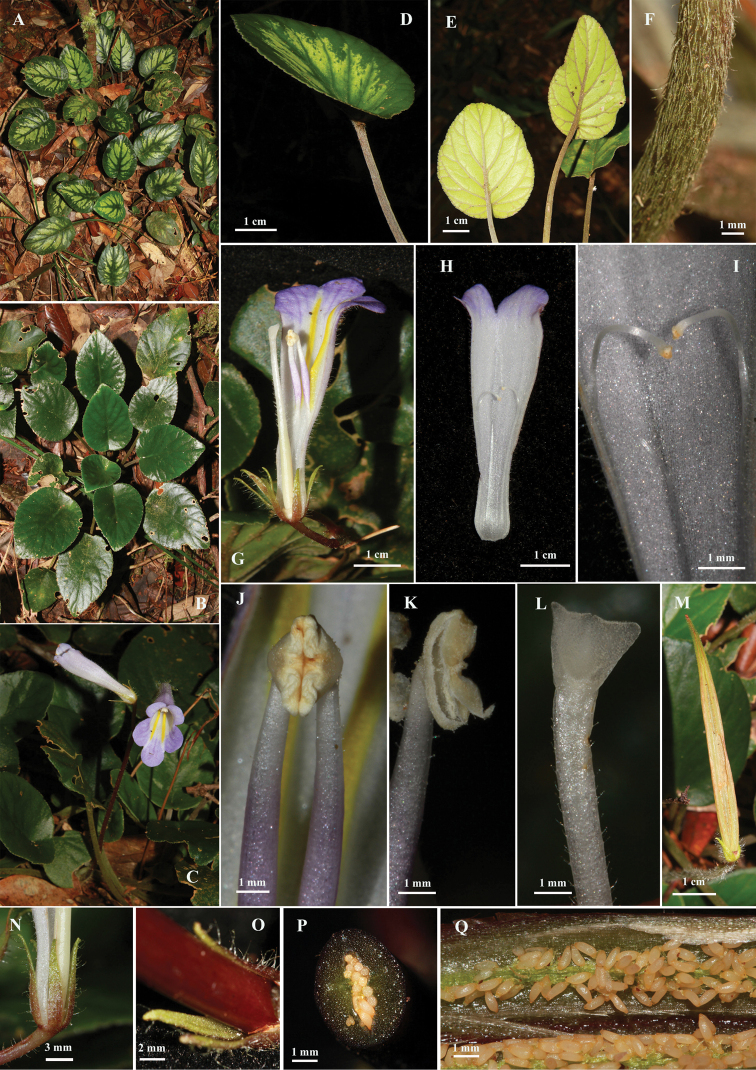
*Primulinascutellifolia***A, B** habit **C** inflorescence **D** leaf blade **E** leaf blade, abaxial surface **F** petiole **G** flower, longitudinal dissection **H** corolla, abaxial lip **I** staminodes **J** anthers **K** opened anther **L** stigma **M** opened fruit **N** calyx lobes, abaxial surface **O** calyx lobes, adaxial surface **P** fruit, cross section **Q** seeds.

#### Description.

Herb, perennial, rosulate, acaulescent. Rhizome terete, woody, to 9 cm long, 3 mm in diameter. Leave 3–13, all basal; petioles cylindrical, appressed downwards tomentose, 6–9 cm long, 0,3 cm in diameter; leaf bade scutellate, 3–5.5 cm long, 2–4 cm wide, adaxially glabrous, shining, plain dark green or dark green with yellowish-greenish spots, leathery, abaxially pale green, reticulate-foveate, sparsely strigose; margins serrate; apex obtuse; base slightly sinuate; venation sunken adaxially, prominent and strigose abaxially; lateral veins 4–6 paired. Inflorescences cymose, axillary, 1–3 flowered. Peduncle reddish brown or greenish, 10–12 cm long, 2–3 mm in diameter, sparsely hirsute. Bracts narrowly triangular, ca 2–3 mm long, ca. 0.5 mm wide at base, same color with peduncle. Calyx 5-lobed from base; lobes equal, lanceolate-oblong 8–10 mm long, 1–2 mm wide, abaxially reddish brown to light green and sparsely glandular hairy, adaxially yellowish green, margin entire, apex acute. Corolla infundibuliform, 4–5 cm long, 1–1.2 cm in diameter at mouth, ca. 0.5 cm in diameter at base, white, violet or white at base and gradually turning to violet towards the apex, outside sparsely glandular hairy, inside smooth and with two yellow stripes on lower part of the corolla. Limb distinctly 2-lipped; upper lip 2-lobed, lobes broadly ovate, 3.5–4 mm long × 7–8 mm wide; lower lip 3-lobed, central lobe orbicular, 7–8 mm long × 7–8 mm wide, lateral ones broadly ovate, 7–8 mm long × 8–9 mm wide. Stamens 2; filaments 29–32 mm long, adnate to the corolla tube base for 16–18 mm, free part 13– 14 mm long and slightly curved, apically sparsely glandular hairy; anthers fused by their entire adaxial surfaces, elliptic, ca. 3 mm long, yellowish. Staminodes 3, linear, translucent; apex capitate, yellowish, glabrous, lateral ones 19–21 mm long, adnate to the corolla for 15–16 mm, free part 4–5 mm long, middle one 16–17 mm long, ca. 1 mm long, adnate to the corolla tube base for 16 mm. Disc ca. 1 mm high, slightly 5-lobed. Ovary linear, 3–3.5 cm long, ca. 2 mm in diameter at base, glabrous on basal 1/2, glandular hairy on apical 1/2. Stigma of only lower lip developing, translucent white, trapeziform, finely hairy, with emarginated apex. Capsule linear, slightly falcate, oblique in relation to the pedicel, reddish brown or greenish and turning to light yellowish, sparsely hairy on the apical part, 65–70 mm long, 5–7 mm in diameter, opening along the dorsal side. Seeds long ellipsoid, translucent brownish.

#### Phenology.

Flowering was found in August to November and fruiting in September to January.

#### Etymology.

The specific epithet is derived from its special scutellate leaves.

#### Vietnamese names.

Báo xuân đón lộc.

#### Distribution and habitat.

*Primulinascutellifolia* is currently only known from the type location. It grows scattered on humid fertile soils in the evergreen broadleaf forest at elevations of 1,450 to 1,950 m. Our surveys throughout forests of Khanh Hoa and Lam Dong Provinces, which have now been ongoing for more than ten years, confirm its distribution is confined to the eastern slopes of the Hon Giao Range. This is a locally endemic plant.

#### Preliminary conservation status.

The plant has been recorded in one population at the type location, with Extent of Occurrence <100 km^2^ that is impacted by continued logging and not effectively protected. Therefore, we suggest the species to be categorized as Critically Endangered (A1a or B1a,b) (IUCN Standards and Petitions Subcommittee 2019).

#### ﻿Discussion

**Figure 2.**
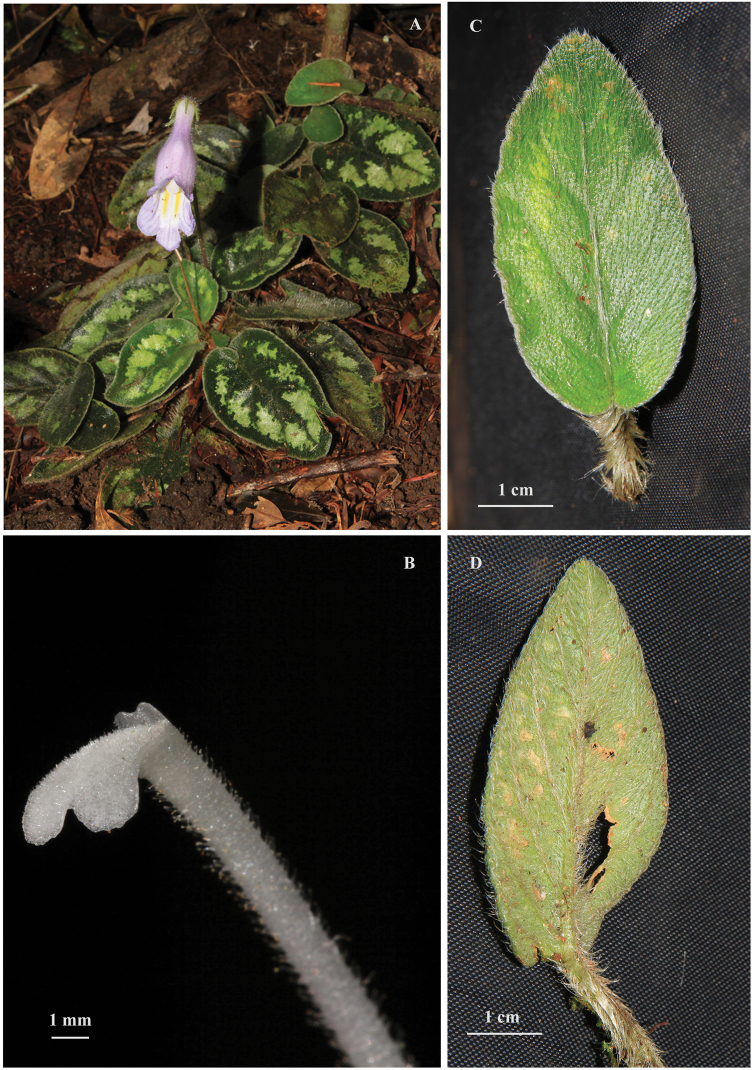
*Primulinaannamensis***A** habit **B** stigma **C** leaf blade, adaxial surface **D** leaf blade, abaxial surface.

*Primulinascutellifolia* is unique in the genus by its scutellate leaves. It may be confused with *P.annamensis* (Figure 2) which has similar general shapes, sizes and colours of habit, inflorescence, flower, and leaf (Pellegrin 1930; Wood 1974; Pham-Hoang 1993; Pham-Hoang 2003; Weber et al. 2011; Vu 2017). However, the latter taxon can be distinguished from our species in having abaxially and adaxially denser silky leaves with obviously cordate base, flat blade and entire or crenate margins, denser pilose petioles, denser glandular hairs on more than apical 2/3 of the gynoecium, bi-lipped stigma and bifid stigma lips with round lobes (Table 1). Both species sometimes grow sympatrically but the latter is much more abundant. In dried specimens, the leaves of the new species look somehow subpeltate, which may be reminiscent of those in *Deinostigmatamiana* (B.L.Burtt) D.J.Middleton & H.J.Atkins from northern Vietnam (Burtt 1999; Möller et al. 2016), but the latter is distinguishable by its slightly peltate leaves with hairs on both surfaces, short petioles, hooked hairs on pedicel and 4–9-flowered inflorescences. The scutellate leaf blades, often with yellowish-greenish spots and beautiful flowers of the new taxon, render it an ornamental plant of great potential.

**Table 1.** Key morphological differences between *Primulinaannamensis* and *P.scutellifolia*.

Characters	*P.annamensis*	*P.scutellifolia*
Petiole	densely pilose	appressed downwards tomentose
Lamina	flat, with obviously cordate base and entire or crenate margins, abaxially and adaxially densely silky	scutellate, with slightly sinuate base and serrate margins, adaxially glabrous, abaxially sparsely strigose
Gynoecium	densely glandular hairy on apical >2/3	glandular hairy on apical 1/2
Stigma	bi-lipped; lips bifid with round lobes	upper lip absent; lower lip with slightly emarginated truncate apex

## Supplementary Material

XML Treatment for Primulina scutellifolia
